# The Beneficial Effects of Probiotics via Autophagy: A Systematic Review

**DOI:** 10.1155/2021/2931580

**Published:** 2021-12-03

**Authors:** Marzieh Nemati, Gholamhossein Ranjbar Omrani, Bahareh Ebrahimi, Nima Montazeri-Najafabady

**Affiliations:** ^1^Endocrinology and Metabolism Research Center, Shiraz University of Medical Sciences, Shiraz, Iran; ^2^Geriatric Research Center, Shiraz University of Medical Sciences, Shiraz, Iran; ^3^Biotechnology Research Center, Shiraz University of Medical Sciences, Shiraz, Iran

## Abstract

Probiotics are living microorganisms increasingly used to treat or modulate different diseases or disorders because of their benefits and also low adverse reaction, and their positive and protective effects on various cells and tissues have been reported. The mechanisms by which probiotics exert their beneficial effects in different cells and tissues were investigated, and autophagy is one of the main mechanisms to induce their positive effects. Autophagy is a conserved process that occurs in all eukaryotic cells and plays an essential role in homeostasis and cell survival by degrading damaged and dysfunctional intracellular organelles. On the other hand, the role of autophagy is diverse in different tissues and situations, and cell death derived from autophagy has been observed in some cells. This search was done in PubMed, WOS, and Scopus using the keywords probiotic, microbiota, and autophagy. The search strategy was focused on the in vitro and animal model studies, and the included filters were English language publications and full-text articles (by June 2020). Studies that investigated other underlying mechanisms except autophagy were excluded. Among more than 105 papers, 24 studies were considered eligible for more evaluation. The obtained results indicated that most studies were performed on intestinal cell lines or tissue compared with other types of cell lines and tissue. This review article discusses our current understanding of the probiotic effects through autophagy in different cell lines and tissues that would be a useful guide to daily and clinical usage of these living microorganisms, but despite promising results of this systematic review, further studies need to assess this issue. This systematic review has demonstrated that autophagy is an effective mechanism in inducing beneficial effects of probiotics in different tissues.

## 1. Background

Probiotics are living microorganisms whose extraordinary and protective effects on various cells and tissues have been described [1]. For example, various studies have shown that administrating adequate doses of probiotics protects the heart against damage [2–4]; increases metabolism [5]; modulates immune system function [3, 6]; increases the survival rate of piglets [7]; improves growth performance, immune status, and gut health of broilers [8]; and contributes to the gastrointestinal health condition [6, 9]. These microorganisms also prevent the pathogen infection in vitro and in vivo conditions [10–12], reduce free radical damage, and prevent tumor progression.

Different pathways have been suggested regarding these organisms' mechanism, but one of their most attractive mechanisms is their effect on autophagy [13, 14]. Autophagy is a highly conserved catabolic process that occurs in all eukaryotic cells [15, 16] and plays an essential role in homeostasis [17] and cell survival [18] by degrading damaged and dysfunctional intracellular organelles [16] and microbial invaders [19]. Therefore, autophagy is considered as a cell basal function on physiologic conditions [20, 21]. On the other hand, the role of autophagy is diverse in different tissues and situations, and autophagy-dependent cell death has been observed in some cells following overloaded autophagy [22, 23], and in the same context, the role of impaired autophagy has been shown in some diseases such as inflammatory bowel disease (IBD), necrotizing enterocolitis (NEC), tuberculosis neurodegeneration, and aging [13]. Overall, it is unclear whether autophagy is a protective response or a deleterious process, but it is clear that uncontrolled autophagy can lead to adverse effects in certain pathological conditions, ultimately causing cell death [13]. There are three known pathways for autophagy processes including chaperone-mediated autophagy (CMA), macroautophagy, and microautophagy. CMA occurs only in mammalian cells and plays a key role in the degradation of single, soluble proteins. In contrast, macro- and microautophagies occur in a wide range of eukaryotes including mammals, plants, and fungi and lead to the degradation of portions, which may include cell organelles [24].

Different proteins are involved in autophagy pathways; for instance, some involving key proteins in the CMA are members of the Hsp70 family of chaperones in the cytosol (Hsc70), the lysosomal lumen (Hsc73), and the lysosomal membrane protein LAMP-2A [25]. Atg genes, Beclin, and LC3 are the most important proteins involved in macro- and microautophagy pathways [26].

The mechanisms by which probiotics increase protective effects in different tissues have been discovered through both in vitro studies and in vivo animal models. In this study, we tried to review the autophagic mechanism of probiotic microbes by which they improve health with emphasis on recent discoveries in the field.

## 2. Search and Study Selection

Keywords and abstract terms included ((probiotic [Title/Abstract] OR microbiota [Title/Abstract])) AND (autophagy [Title/Abstract]). The search strategy was applied to PubMed database, WOS (Web of Science), and Scopus, being focused on the in vitro and animal model studies, and the included filter was English language publications (by June 2020). Abstracts not published as full manuscripts, reviews, or probiotics inducing a mechanism aside from autophagy were excluded. Data were collected from the full-text articles as follows: (i) the target tissue, (ii) type of used probiotics, (iii) type of the study (in vitro or in vivo), and (iv) the obtained results.

## 3. Results

**Figure 1:**
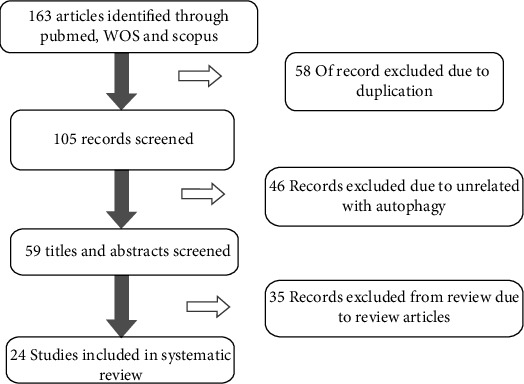
Literature search and study selection flowchart.

Search results and characteristics of included articles yielded 163 studies. Among them, 105 papers received all inclusion criteria and were selected after removing duplications. After the initial reading of articles, 35 papers were reviewed and excluded (Figure 1); among them, 12 articles had investigated the effects of probiotics through induction of autophagy in vitro (Table 1) and 16 articles were related to the effects of probiotics on an animal model organ via autophagy (Table 2). These studies' extraction data are shown in Tables 1 and 2. These results showed that probiotic benefits via autophagy induction have been studied in different cell lines; intestinal epithelial cell (IEC) lines were used in 6 studies and intestinal organoids were used in one study, whereas one study used human colon cell line; 3 papers used human monocyte-derived macrophage (HMDM), macrophage cell line (Raw264.7), and mesenteric lymph node cell (MLNC); and one article used bone marrow cells (BMCs). Analysis of target tissue showed that probiotic effects on autophagy were studied in multiple tissues; different parts of the intestine were investigated in 8 studies, and with regard to human placenta, Alzheimeric mice, mouse pharyngeal and kidney epithelium, mouse liver tissue, and rat cardiac tissue, each have been examined only in one study. In addition, 2 studies investigated fish autophagy induced by probiotics. Different probiotics were used in these studies, and their benefits on different cells and tissues through induction of autophagy were investigated. Below, we discuss these studies according to their effects on autophagy induction.

### 3.1. Effects of Probiotic-Induced Autophagy on the Various Cell Lines

Treating human colonic Caco2 BBE [27, 28] and rat jejunum IEC 18 cells [27] with conditioned media from *Bifidobacterium breve* (BB-CM) [27–29] or other intestinal bacteria (*Lactobacillus plantarum* and *Lactobacillus rhamnosus* GG) [27] could induce and activate autophagy in intestinal epithelial cells to promote their survival and other beneficial effects during stress. Other investigations on IEC-6 cells, T84 cells, and IEC-18 cells have revealed that treating with *Bacillus amyloliquefaciens SC06* and *Bifidobacteria* alleviated apoptosis via p38-mediated autophagy [30], improved intestinal mucus layer function [29], and provided enteroprotection against LPS-induced intestinal epithelial toxicity [31].

Lu et al. demonstrated that using probiotic lactic acid bacteria (LAB) had activated autophagic responses on human cell line HCT116 or intestinal organoids [32]. The result of another study also had shown the antitumor effects of LAB HT-29 on colon cancer cells through the activation of autophagy [14]. Wu et al. found that *Bacillus amyloliquefaciens SC06* (*Ba*) via inducing autophagy provides antibacterial activity against *Escherichia coli* (E. *coli*) in the murine macrophage cell line (RAW264.**7** cells) [33]. *Lactobacillus brevis BGZLS10-17* via autophagy had strong immunoregulatory effects on mesenteric lymph node cells (*MLNC*) [34]. Also, treatment of human monocyte-derived macrophages (HMDMs) with two strains of *LAB* induced autophagy, leading to destruction of intracellular Mycobacterium tuberculosis (Mtb) and survival of mononuclear phagocytes [35]. Zaylaa et al. reported the anti-inflammatory abilities of *lactobacilli*, through inducing autophagy, on bone marrow cells derived from autophagy-related 16-like 1-deficient mice [36].

### 3.2. Effects of Probiotic-Induced Autophagy on Various Tissues

#### 3.2.1. Gastrointestinal Tissue

The benefits of using probiotics in the prevention and treatment of gastrointestinal diseases have received considerable attention in recent years. Although several mechanisms are involved in this effectiveness, our intent is to paint a broad picture of the autophagy mechanism and to highlight recent discoveries in this rapidly expanding field. So different studies have reported beneficial effects of various probiotics on intestinal tissue in both normal and damaged conditions via the autophagy mechanism. For example, Inaba et al. have described the induction of autophagy-dependent cell protection after using Gram-positive bacterium *Bifidobacterium breve* (*Bb-CM*) in mice conditionally deficient in intestinal epithelial cell Atg7 [28]. Other studies have reported that administration of *Lactobacillus rhamnosus GG* (*LGG*) alone or in combination with *Lactobacillus reuteri ZJ617* (*ZJ617*) reduced autophagy marker expression and light chain 3 (LC3) activity during viral gastroenteritis and physical barrier integrity which prevent tissue damage and maintain gut homeostasis [13, 37]. Engevik et al. revealed that *B. dentium* enhanced the intestinal mucus layer and goblet cell function via upregulating gene expression and autophagy signaling pathways [29]. Also, treating intestinal epithelial VDR (vitamin D receptor) conditional knockout (VDR*Δ*IEC) mice with *lactic acid bacteria* (*LAB*) could activate autophagy responses and inhibit the inflammation [32]. Administration of *Bacillus amyloliquefaciens* (*Ba*) to the piglet or soybean meal fermented to weaned piglets could significantly improve the growth performance via autophagy [38, 39]. Wu et al. described that orally administered *Bacillus SC06* can alleviate oxidative stress-induced disorders in rat jejunum by triggering autophagy [30].

#### 3.2.2. Productive Tissue and Development

Probiotics have been identified to play important roles in many biological systems, including growth, development, and reproduction [21]. Its mechanism is not yet entirely clear, but autophagy seems to play a significant role in inducing beneficial effects of probiotics. A study conducted by Yang et al. indicated that probiotic supplementation may induce a reduction in the autophagy-related protein Beclin1 at the mRNA level in the placentas undergoing spontaneous delivery [40]. Another study showed that parentally administered *Lactobacillus rhamnosus* can modulate some physiological processes involved in zebrafish embryo development [21]. Gioacchini et al. demonstrated that *L. rhamnosus IMC 501* can regulate ovary physiology in zebrafish by inhibiting follicular apoptosis and improving follicular survival [41].

#### 3.2.3. Cardiovascular Tissue

Further evidence has shown a biologic effect of probiotics on the heart [2, 3]. Several studies have reported the positive role of probiotics through the regulation of autophagy expression [13, 14]. Lai et al. showed that the level of autophagy pathway proteins significantly reduced after oral administration of probiotics, and this can attenuate on cardiomyocyte fibrosis in obese rats [42].

#### 3.2.4. Neural Tissue

Recent studies have highlighted a microbiome role on regulating multiple neurochemical pathways through the gut-brain axis [43, 44]. Following this, beneficial effects of some probiotics on CNS-related diseases, such as multiple sclerosis, cognitive deficits, and stress-derived pathologies, have been recently reported [45–47]. Results of a study that was conducted by Bonfili et al. have shown partial restoration of impaired neuronal proteolytic pathways (autophagy), reduction in brain damage, and cognitive decline in 3xTg-Alzheimeric mice treated with *SLAB51* probiotics [48].

#### 3.2.5. Kidney Tissue

Recently, a connection between the intestine and kidneys has been suggested [49, 50]. Several studies have shown that modification of microbiota composition could have an effect on glomerulopathy outcome [51, 52]. For example, Andrade-Oliveira et al. found that acetate treatment could reduce apoptosis, increase autophagy and tubular proliferating cells, and prevent AKI induced by ischemia-reperfusion [53].

#### 3.2.6. Liver Tissue

Several studies have shown the probiotic benefits on various hepatic diseases through different pathways such as inhibiting TLR4-mediated endotoxin and TNF-*α* production [54], suppressing the MAPK and NF-*κ*B signaling pathways [55], and upregulating nuclear factor erythroid 2-related factor 2 and its downstream antioxidative genes [56]. Cui et al. found that oral inoculation culture supernatant of *Lactobacillus reuteri* (*ZJ617s*) can ameliorate LPS-induced liver injury through suppression of autophagy in mice [57].

#### 3.2.7. Other Tissues

One study investigated the effects of probiotics on pharynx tissue and demonstrated that AR809 administration through reducing the production of inflammatory mediators and elevating the autophagic proteins showed protective effects on pharyngitis and may be used in preventing pharyngitis and other inflammatory diseases [58].

## 4. Discussion

This study is the first systematic review about probiotic benefits with emphasis on recent discoveries about the autophagy mechanisms. This review results were based on in vitro investigations and animal studies. The various types of probiotics used in these studies and different evaluation methods make it interesting and beneficial to compare their obtained results with each other. In most studies, *Lactobacillus* and *Bacillus* were used in both in vitro and in vivo conditions for assessing the beneficial effects of probiotics in different tissues. Results of several studies indicated that probiotic administration induces, regulates, or modulates autophagy through activating different signaling pathways, in particular micro- and macroautophagy pathways by increasing expression of the autophagy-related (ATG) genes such as Atg5, Atg7 [28, 34], Atg5-Atg12-Atg16 complex [33], Beclin1 (autophagy marker protein level) [14, 33], and GRP78 [14], via p38-mediated [30] or via mTOR signaling pathway [37] which could promote survival [28]; improve growth performance [38, 39]; prevent virus-induced tissue damage [13]; promote and maintain gut homeostasis [37]; protect intestinal mucus layer function [29]; exert anti-inflammatory activity [32] in intestinal tissue, intestinal cell line, or other cell lines [33, 34]; and also induce death in tumor cells [14].

**Figure 2:**
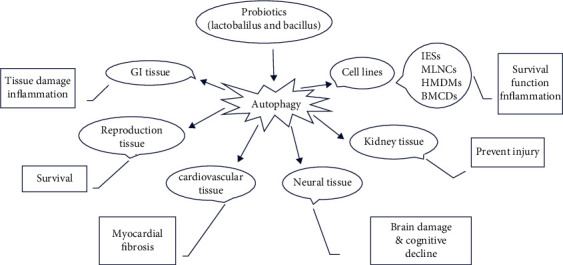
Schematic summary of probiotics effects via autophagy on different cell lines and tissues.

Several studies reported that probiotic administration through increasing autophagy pathway activity such as enhancing the level of Beclin1 [48, 53] and LC3 development [21, 53] led to reduced brain damage and cognitive decline in mice [48], improved embryonic development [21], and improved renal dysfunction [53]. However, other studies showed that probiotic supplementation via reducing or suppressing autophagy pathways could prevent the occurrence of placenta-derived diseases [40] or exert beneficial effects on the liver tissue [57]. Below, the schematic figure regarding the summary of probiotic effects on different cell lines and tissues via autophagy that was discussed in this systematic review is given (Figure 2).

Although several studies have been performed on the beneficial effects of probiotics through autophagy, further and more precise assessment is needed to confirm the importance of the autophagy mechanism in the incidence of the favorable effects of probiotics.

## 5. Conclusion

In summary, this systematic review has demonstrated that autophagy is an effective mechanism in inducing beneficial effects of probiotics in different tissues. Autophagy appeared to be the main mechanism in the intestinal tissue structure and function in both normal and disease conditions. There is little evidence about the beneficial effects of probiotics via autophagy in the treatment or prevention of disorders in other tissues. However, a few numbers of studies have examined and showed this issue in other tissues; further studies are required to determine the importance of autophagy in exerting beneficial effects of probiotics in different cells and tissues.

## Figures and Tables

**Table 1:** The effects of probiotics through induction of autophagy in vitro.

Probiotics	Cell lines	Outcomes	References
Bifidobacterium breve Lactobacillus plantarum Lactobacillus rhamnosus (GG)	Human intestinal epithelial cells	Probiotics induce autophagy in gut epithelial cells that have beneficial clinical effects attributed to a healthy enteric	[25]
Bifidobacteria	Rat intestinal epithelial cell line	Probiotics stimulated an autophagy response in IEC, thereby maintaining balanced crosstalk with intestinal epithelia	[35]
Bifidobacterium breve	Mice intestinal epithelial cells	Probiotics increase expression of the autophagy proteins and promote their survival during stress	[26]
Bifidobacteria	Rat intestinal epithelial cell line	Probiotic treatment through inhibiting LPS-induced autophagy provides enteroprotection and maintains gut homeostasis	[29]
B. dentium	Mice intestinal cells	Probiotics via upregulation of gene expression and autophagy signaling pathways, net increased production	[27]
Bacillus SC06 or SC08	Rat intestinal epithelial cell line	Attenuates oxidative stress-induced intestinal injury via p38-mediated autophagy	[28]
Lactic acid bacteria (LAB)	Mice intestinal epithelial cells	LAB exert anti-infection and anti-inflammation effects through inducing autophagy	[30]
LAB	Human colon cancer cells	Probiotic treatment exert antitumourigenic via the activation of autophagic cell death in cancer cells	[12]
Bacillus amyloliquefaciens SC06	Murine macrophage cell line RAW264.7	Probiotics-mediated autophagy, prevent bacterial infection in intestine	[31]
Lactobacillus brevis BGZLS10-17	MLNC	The immunoregulatory effects of *Lactobacillus brevis* BGZLS10-17 via ATG5-dependent autophagy in MLNC	[32]
Lactic acid bacteria (LAB)	HMDMs	LAB enhance the autophagic ability of mononuclear phagocytes in response to Mtb antigen	[33]
Lactobacilli and Bifidobacteria	BMCD from autophagy protein-deficient mice	Probiotics via autophagy induce immune-regulatory responses	[34]

BMCD: bone marrow cell derived; HMDMs: human monocyte-derived macrophages; MLNC: mesenteric lymph node cells.

**Table 2:** The effects of probiotics on different tissues in animal models via autophagy.

Probiotics	Tissue/disease	Outcomes	References
Bifidobacterium breve	Intestine	Probiotics modify protein degradation programs within the intestinal epithelial cells to promote their survival during stress.	[26]
Bacillus	Intestine	Probiotics improved growth performance via increasing intestinal autophagy.	[36]
Lactobacillus	Intestinal injury gastroenteritis	Probiotics reduced autophagy marker expression to normal levels and partially prevented virus-induced tissue damage.	[11]
L. plantarum, B. and S. cerevisiae	Intestine	Probiotic feeding improved the growth, immune function, and intestinal health in weaned piglets.	[37]
Bacillus (SC06 or SC080	Intestine	*Bacillus SC06* alleviated oxidative stress-induced disorders and apoptosis via p38-mediated autophagy.	[28]
Lactobacillus	Intestine	Probiotics supplementation protected LPS-induced intestinal barrier dysfunction via attenuating apoptosis and autophagy via mTOR signaling pathway.	[35]
B. dentium	Intestine	Probiotics enhanced the intestinal mucus layer and goblet cell function via upregulation of gene expression and autophagy signaling pathways.	[27]
LAB	Intestine	Probiotics caused anti-infection and anti-inflammation via inducing autophagy.	[30]
Lactobacillus rhamnosus, Pediococcus acidilactici, Bifidobacterium adolescentis	Cardiac tissue	Oral administration of probiotics provided cardiac protection via regulation of fibrosis and autophagy.	[40]
SLAB51	Alzheimer disease	Prebiotic treatment by activating autophagy decreased the brain damage and cognitive decline in Alzheimeric mice.	[46]
Short-chain fatty acids (SCFAs) are produced by the intestinal microbiota	Kidney	*SCFAs* improved the renal dysfunction caused by injury. This protection was partially associated with an increase in autophagy.	[51]
ZJ617	Liver	*ZJ617s* exerted beneficial effects on the mouse liver through suppression of hepatic TLR4/MAPK/NF-*κ*B activation and autophagy.	[55]
Golden bifid	Placenta	Oral supplementation with golden bifid induced placental protection via reducing the autophagy-related protein Beclin1.	[38]
Lactobacillus rhamnosus	Zebrafish	Parental *Lactobacillus rhamnosus* administration can modulate important physiological processes involved in zebrafish embryo development.	[19]
Lactobacillus rhamnosus	Ovarian follicles	Probiotics modulated the balance between apoptosis and autophagy and improved the follicular survival.	[39]
Lactobacillus salivarius AR809	Pharyngeal epithelium	AR809 prevents S. aureus-induced pharyngeal inflammatory response, possibly by regulating mTOR signaling pathway-related autophagy.	[56]

SLAB51: a formulation made of nine live bacterial strains [*Streptococcus thermophilus*, *Bifidobacteria* (*B. longum*, *B. breve*, *B. infantis*), *Lactobacillus* (*L. acidophilus*, *L. plantarum*, *L. paracasei*, *L. delbrueckii* subsp. *bulgaricus*, *L. brevis*)]; LAB: lactic acid bacteria; ZJ617: *Lactobacillus reuteri*.

## Data Availability

The datasets used and/or analyzed during the current study are available from the corresponding author on reasonable request.

## References

[1] Rehan I. F., Youssef M., Abdel-Rahman M. A. M. (2020). The impact of probiotics and egg yolk IgY on behavior and blood parameters in a broiler immune stress model. *Frontiers in Veterinary Science*.

[2] Gan X. T., Ettinger G., Huang C. X. (2014). Probiotic administration attenuates myocardial hypertrophy and heart failure after myocardial infarction in the rat. *Circulation: Heart Failure*.

[3] Costanza A. C., Moscavitch S. D., Neto H. C. F., Mesquita E. T. (2015). Probiotic therapy with Saccharomyces boulardii for heart failure patients: a randomized, double-blind, placebo-controlled pilot trial. *International Journal of Cardiology*.

[4] DiRienzo D. B. (2014). Effect of probiotics on biomarkers of cardiovascular disease: implications for heart-healthy diets. *Nutrition Reviews*.

[5] Gorenjak M., Gradišnik L., Trapečar M. (2014). Improvement of lipid profile by probiotic/protective cultures: study in a non-carcinogenic small intestinal cell model. *The New Microbiologica*.

[6] Prakash S., Tomaro-Duchesneau C., Saha S. (2011). The gut microbiota and human health with an emphasis on the use of microencapsulated bacterial cells. *Journal of Biomedicine and Biotechnology*.

[7] Wenfeng S., Juan Z., Yongjian G., Hui D., Liangwei Q. (2015). Application of probiotics in pig industry as a substitute of antibiotics. *Animal Husbandry and Feed Science*.

[8] Abdel-Latif M. A., Abd El-Hack M. E., Swelum A. A. (2018). Single and combined effects of *Clostridium butyricum* and *Saccharomyces cerevisiae* on growth indices, intestinal health, and immunity of broilers. *Animals*.

[9] Whelan K., Quigley E. M. (2013). Probiotics in the management of irritable bowel syndrome and inflammatory bowel disease. *Current Opinion in Gastroenterology*.

[10] Lebeer S., Vanderleyden J., De Keersmaecker S. C. (2008). Genes and molecules of *Lactobacilli* supporting probiotic action. *Microbiology and Molecular Biology Reviews*.

[11] Candela M., Perna F., Carnevali P. (2008). Interaction of probiotic Lactobacillus and Bifidobacterium strains with human intestinal epithelial cells: adhesion properties, competition against enteropathogens and modulation of IL-8 production. *International Journal of Food Microbiology*.

[12] Zhou D., Zhu Y.-H., Zhang W. (2015). Oral administration of a select mixture of *Bacillus* probiotics generates Tr1 cells in weaned F4ab/acR^−^ pigs challenged with an F4^+^ ETEC/VTEC/EPEC strain. *Veterinary Research*.

[13] Wu S., Yuan L., Zhang Y. (2013). Probiotic *Lactobacillus rhamnosus* GG mono-association suppresses human rotavirus-induced autophagy in the gnotobiotic piglet intestine. *Gut Pathogens*.

[14] Kim Y., Oh S., Yun H., Oh S., Kim S. (2010). Cell-bound exopolysaccharide from probiotic bacteria induces autophagic cell death of tumour cells. *Letters in Applied Microbiology*.

[15] Mizushima N. (2011). Autophagy in protein and organelle turnover. *Cold Spring Harbor Symposia on Quantitative Biology, 2011*.

[16] Kang R., Zeh H., Lotze M., Tang D. (2011). The Beclin 1 network regulates autophagy and apoptosis. *Cell Death and Differentiation*.

[17] Yang Z., Klionsky D. J. (2009). An Overview of the Molecular Mechanism of Autophagy. *Autophagy in infection and immunity*.

[18] Levine B., Mizushima N., Virgin H. W. (2011). Autophagy in immunity and inflammation. *Nature*.

[19] Shi C.-S., Shenderov K., Huang N.-N. (2012). Activation of autophagy by inflammatory signals limits IL-1*β* production by targeting ubiquitinated inflammasomes for destruction. *Nature Immunology*.

[20] Jiang Y., Gao M., Wang W. (2015). Sinomenine hydrochloride protects against polymicrobial sepsis via autophagy. *International Journal of Molecular Sciences*.

[21] Miccoli A., Gioacchini G., Maradonna F., Benato F., Skobo T., Carnevali O. (2015). Beneficial bacteria affect *Danio rerio* development by the modulation of maternal factors involved in autophagic, apoptotic and dorsalizing processes. *Cellular Physiology and Biochemistry*.

[22] Eskelinen E.-L., Saftig P. (2009). Autophagy: a lysosomal degradation pathway with a central role in health and disease. *Biochimica et Biophysica Acta (BBA)-Molecular Cell Research*.

[23] Mizushima N., Levine B., Cuervo A. M., Klionsky D. J. (2008). Autophagy fights disease through cellular self-digestion. *Nature*.

[24] Kiššová I. B., Salin B., Schaeffer J., Bhatia S., Manon S., Camougrand N. (2007). Selective and non-selective autophagic degradation of mitochondria in yeast. *Autophagy*.

[25] Dice J. F. (1990). Peptide sequences that target cytosolic proteins for lysosomal proteolysis. *Trends in Biochemical Sciences*.

[26] Mehrpour M., Esclatine A., Beau I., Codogno P. (2010). Autophagy in health and disease. 1. Regulation and significance of autophagy: an overview. *American Journal of Physiology-Cell Physiology*.

[27] Inaba Y., Fujiya M., Musch M. W., Boone D. L., Kohgo Y., Chang E. B. (2011). Activation of intestinal epithelial autophagy as a potential and novel mechanism of probiotic action in the gut. *Gastroenterology*.

[28] Inaba Y., Ueno N., Numata M. (2016). Soluble bioactive microbial mediators regulate proteasomal degradation and autophagy to protect against inflammation-induced stress. *Physiology*.

[29] Engevik M. A., Luk B., Chang-Graham A. L. (2019). *Bifidobacterium dentium* fortifies the intestinal mucus layer via autophagy and calcium signaling pathways. *MBio*.

[30] Wu Y., Wang B., Xu H. (2019). Probiotic Bacillus attenuates oxidative stress-induced intestinal injury via p38-mediated autophagy. *Frontiers in Microbiology*.

[31] Han C., Ding Z., Shi H., Qian W., Hou X., Lin R. (2016). The role of probiotics in lipopolysaccharide-induced autophagy in intestinal epithelial cells. *Cellular Physiology and Biochemistry*.

[32] Lu R., Shang M., Zhang Y.-G. (2020). Lactic acid bacteria isolated from Korean kimchi activate the vitamin D receptor–autophagy signaling pathways. *Inflammatory Bowel Diseases*.

[33] Wu Y., Wang Y., Zou H. (2017). Probiotic *Bacillus amyloliquefaciens* SC06 induces autophagy to protect against pathogens in macrophages. *Frontiers in Microbiology*.

[34] Bajić S. S., Đokić J., Dinić M. (2020). GABA potentiate the immunoregulatory effects of Lactobacillus brevis BGZLS10-17 via ATG5-dependent autophagy in vitro. *Scientific Reports*.

[35] Ghadimi D., de Vrese M., Heller K. J., Schrezenmeir J. (2010). Lactic acid bacteria enhance autophagic ability of mononuclear phagocytes by increasing Th1 autophagy-promoting cytokine (IFN-*γ*) and nitric oxide (NO) levels and reducing Th2 autophagy-restraining cytokines (IL-4 and IL-13) in response to Mycobacterium tuberculosis antigen. *International Immunopharmacology*.

[36] Zaylaa M., Alard J., Al Kassaa I. (2019). Autophagy: a novel mechanism involved in the anti-inflammatory abilities of probiotics. *Cellular Physiology and Biochemistry*.

[37] Cui Y., Liu L., Dou X. (2017). *Lactobacillus reuteri* ZJ617 maintains intestinal integrity via regulating tight junction, autophagy and apoptosis in mice challenged with lipopolysaccharide. *Oncotarget*.

[38] Wang Y., Wu Y., Wang B. (2017). Effects of probiotic *Bacillus* as a substitute for antibiotics on antioxidant capacity and intestinal autophagy of piglets. *AMB Express*.

[39] Zhu J., Gao M., Zhang R. (2017). Enhanced heterologous protein productivity by genome reduction in Lactococcus lactis NZ9000. *Microbial Cell Factories*.

[40] Yang P., Li Z., Tye K. D. (2020). Effects of an orally supplemented probiotic on the autophagy protein LC3 and Beclin1 in placentas undergoing spontaneous delivery during normal pregnancy. *BMC Pregnancy and Childbirth*.

[41] Gioacchini G., Dalla Valle L., Benato F. (2013). Interplay between autophagy and apoptosis in the development of *Danio rerio* follicles and the effects of a probiotic. *Reproduction, Fertility and Development*.

[42] Lai C.-H., Tsai C.-C., Kuo W.-W. (2016). Multi-strain probiotics inhibit cardiac myopathies and autophagy to prevent heart injury in high-fat diet-fed rats. *International Journal of Medical Sciences*.

[43] Bhattacharjee S., Lukiw W. J. (2013). Alzheimer's disease and the microbiome. *Frontiers in Cellular Neuroscience*.

[44] Li P. C., Yang Y. C., Hwang G. Y., Kao L. S., Lin C. Y. (2014). Inhibition of reverse-mode sodium-calcium exchanger activity and apoptosis by levosimendan in human cardiomyocyte progenitor cell-derived cardiomyocytes after anoxia and reoxygenation. *PLoS One*.

[45] Duncan S. H., Flint H. J. (2013). Probiotics and prebiotics and health in ageing populations. *Maturitas*.

[46] Camfield D. A., Owen L., Scholey A. B., Pipingas A., Stough C. (2011). Dairy constituents and neurocognitive health in ageing. *British Journal of Nutrition*.

[47] Hsiao E. Y., McBride S. W., Hsien S. (2013). Microbiota modulate behavioral and physiological abnormalities associated with neurodevelopmental disorders. *Cell*.

[48] Bonfili L., Cecarini V., Berardi S. (2017). Microbiota modulation counteracts Alzheimer's disease progression influencing neuronal proteolysis and gut hormones plasma levels. *Scientific Reports*.

[49] Anders H.-J., Andersen K., Stecher B. (2013). The intestinal microbiota, a leaky gut, and abnormal immunity in kidney disease. *Kidney International*.

[50] Vitetta L., Gobe G. (2013). Uremia and chronic kidney disease: the role of the gut microflora and therapies with pro- and prebiotics. *Molecular Nutrition & Food Research*.

[51] Vitetta L., Linnane A. W., Gobe G. C. (2013). From the gastrointestinal tract (GIT) to the kidneys: live bacterial cultures (probiotics) mediating reductions of uremic toxin levels via free radical signaling. *Toxins*.

[52] Ramezani A., Raj D. S. (2014). The gut microbiome, kidney disease, and targeted interventions. *Journal of the American Society of Nephrology*.

[53] Andrade-Oliveira V., Amano M. T., Correa-Costa M. (2015). Gut bacteria products prevent AKI induced by ischemia-reperfusion. *Journal of the American Society of Nephrology*.

[54] Wang Y., Liu Y., Kirpich I. (2013). Lactobacillus rhamnosus GG reduces hepatic TNF *α* production and inflammation in chronic alcohol-induced liver injury. *The Journal of Nutritional Biochemistry*.

[55] Hsu T.-C., Huang C.-Y., Liu C.-H., Hsu K.-C., Chen Y.-H., Tzang B.-S. (2017). *Lactobacillus paracasei GMNL-32*, *Lactobacillus reuteri* GMNL-89 and *L. reuteri* GMNL-263 ameliorate hepatic injuries in lupus-prone mice. *British Journal of Nutrition*.

[56] Zhao L., Jiang Y., Ni Y. (2017). Protective effects of Lactobacillus plantarum C88 on chronic ethanol-induced liver injury in mice. *Journal of Functional Foods*.

[57] Cui Y., Qi S., Zhang W. (2019). *Lactobacillus reuteri* ZJ617 culture supernatant attenuates acute liver injury induced in mice by lipopolysaccharide. *The Journal of Nutrition*.

[58] Jia G., Liu X., Che N. (2020). Human-origin *Lactobacillus salivarius* AR809 protects against immunosuppression in S. *aureus*-induced pharyngitis via Akt-mediated NF-*κ*B and autophagy signaling pathways. *Food & Function*.

